# Prevalence of Hepatitis E Virus Infection among Laboratory Rabbits in China

**DOI:** 10.3390/pathogens10060780

**Published:** 2021-06-21

**Authors:** Lin Wang, Chunnan Liang, Xiaobo Li, Ji Wang, Rui Fu, Jin Xing, Jingyi Shu, Chenyan Zhao, Weijin Huang

**Affiliations:** 1 Health Science Center, Department of Microbiology, School of Basic Medical Sciences, Peking University, Beijing 100191, China; 1811210022@pku.edu.can; 2 Institute for Laboratory Animal Resources, National Institutes for Food and Drug Control (NIFDC), Beijing 102629, China; lixiaobo@nifdc.org.cn (X.L.); wangji@nifdc.org.cn (J.W.); wj_nd_jds@sina.com (R.F.); furui78@nifdc.org.cn (J.X.); 3 Institute for Biological Product Control, National Institutes for Food and Drug Control (NIFDC), Beijing 102629, China; zhaochenyan@nifdc.org.cn

**Keywords:** hepatitis E virus, ant-HEV antibody, HEV antigen, laboratory rabbit

## Abstract

Hepatitis E virus (HEV) is zoonotic and the leading cause of acute viral hepatitis worldwide. Rabbit HEV can infect humans and is prevalent globally. It is reported that laboratory rabbits are also naturally infected with HEV. Therefore, it is important to investigate in a large scale the prevalence of HEV in laboratory rabbits. Serum samples were collected from 649 laboratory rabbits of 13 different commercial vendors in Beijing, China, from 2017 to 2019, and anti-HEV and HEV antigen (Ag) were tested. Fecal samples were collected from 50 laboratory rabbits from one of the vendors for HEV RNA detection. Six laboratory rabbits with natural HEV infection were euthanized and their liver, kidney, bile and urine samples were collected for HEV RNA quantification. Liver tissues were subjected to histopathology analysis. The overall positive rates of anti-HEV antibodies and HEV-Ag are 2.6% (15/588) and 7.9% (51/649), respectively. HEV RNA was detected in 12.0% (6/50) of the rabbits. High viral load of HEV RNA was detected in liver and bile samples. Liver inflammation was observed. HEV is circulating in laboratory rabbit population in China. Strict screening is crucial to ensure experimental accuracy and prevent zoonotic transmission to research personnel.

## 1. Introduction

Hepatitis E virus (HEV) is a single-stranded, positive sense RNA virus and the major cause of acute viral hepatitis worldwide. It is estimated that 20 million infections occur annually, leading up to 3 million symptomatic cases and 70,000 deaths [1]. There are four major HEV genotypes (HEV1-4) that are closely related to human infections [2]. HEV1 and HEV2 are endemic in developing countries causing large water-borne outbreaks. HEV3 and HEV4 are zoonotic and infections mainly manifest themselves as sporadic cases via the consumption of contaminated animal meat products [1].

Since the first discovery of HEV in pigs in 1997 [3], HEV has been recognized as an important zoonotic virus with an expanding host range. Domestic pigs [3], wild boars [4] and deer [5] are well-recognized reservoirs for human transmission. However, recent studies discovered that HEV strains originated from rabbits [6,7,8,9], camels [10] and rats [11,12,13] are also capable of causing human hepatitis, posing an increasing threat to people with close contact to these animals.

In 2009, researchers firstly reported in China that rabbit is a novel animal host of HEV harboring a novel HEV genotype, the rabbit HEV [14]. Subsequently, rabbit HEV has been detected in several types of rabbits and hares in many countries, including the USA [15], France [16], Germany [17], Italy [18] and Korea [19]. Later, rabbit HEV was classified as a distant subtype of HEV3, designated as HEV-3ra [20]. Several human cases of HEV-3ra acute or chronic infections have been discovered in Europe, indicating a novel zoonotic threat [6,7,8,9]. Rabbit is not only a common economic animal in meat and fur industries worldwide, but also widely used as laboratory animal in biomedical research. However, HEV is not within the list of excluded pathogens in laboratory rabbit and previous studies have reported naturally occurring HEV infection in laboratory rabbits. HEV RNA was detected in 4.8% and 46.2% of the fecal samples collected from the laboratory rabbits in Beijing [21] and Xi’an City, China [22]. One study further confirmed that the HEV detected in the laboratory rabbit’s feces is infectious by successfully infecting pigs, suggesting potential threat of zoonotic transmission [22]. Besides the discovery in China, past or recent HEV infection has also been detected in the laboratory rabbits in the USA [23] and Korea [24]. Using HEV-infected rabbits in biomedical research may not only confound the results but also leave research personnel to HEV exposure. Therefore, it is of great importance to investigate the prevalence of HEV-3ra infection in laboratory rabbits in a larger scale and extent. In this study, we collected serum samples from 649 laboratory rabbits of 13 different commercial vendors in China from 2017 to 2019, and anti-HEV antibodies and HEV antigen (Ag) were tested. Partial HEV sequences were detected in six rabbits with current HEV infection and the viral load in different tissues and urine were determined.

## 2. Results

### 2.1. Seroprevalence of Anti-HEV Antibodies and HEV-Ag of Laboratory Rabbits

A total number of 649 laboratory rabbits were detected during 2017–2019 in this study and their serum samples were collected (Table 1). Thirty-two serum samples were not sufficient in volume for both anti-HEV antibodies and HEV-Ag detection and only HEV-Ag was tested. The overall positive rates of anti-HEV antibodies and HEV-Ag are 2.6% (15/588) and 7.9% (51/649), respectively. The highest positive rate for ani-HEV antibodies occurred in 2017 (3.1%, 8/258) and for HEV-Ag occurred in 2018 (13.0%, 26/200).

### 2.2. Detection of HEV RNA in Laboratory Rabbits

**Figure 1:**
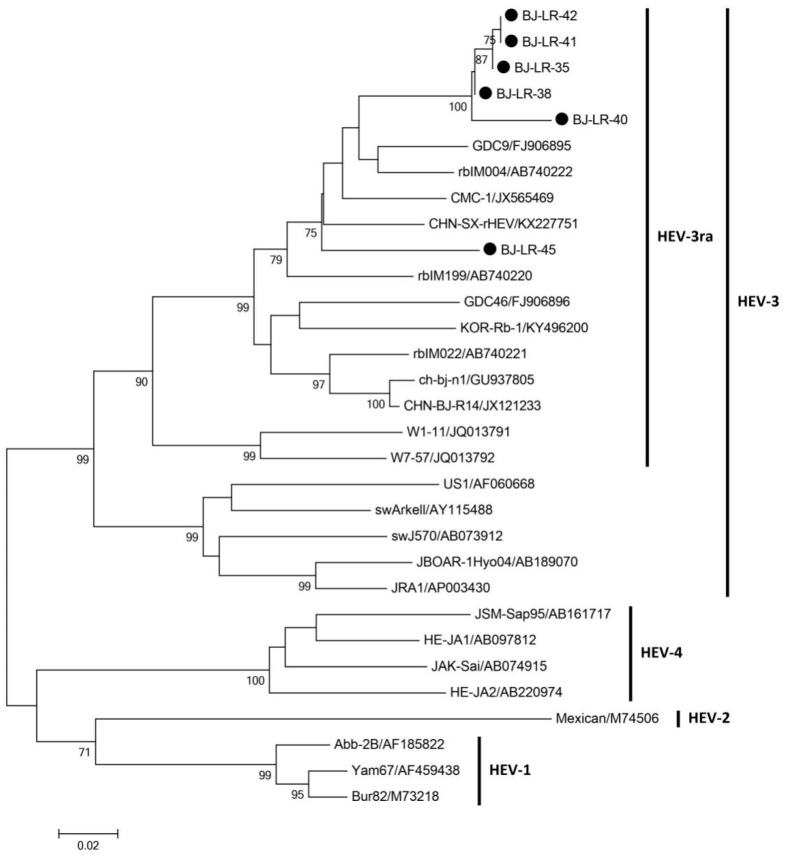
Phylogenetic tree of hepatitis E virus (HEV) isolates obtained from laboratory rabbits. The phylogenetic tree was constructed by using the neighbor-joining method. A partial nucleotide sequence of the open reading frame 2 region and known HEV sequences obtained from GenBank as references. One thousand resampling of the data was used to calculate percentages (values along branches) of tree branches obtained. Black circles indicate laboratory rabbit isolates sequenced during the current study with GenBank accession numbers MZ032036-MZ032041. GenBank accession nos. of all reference sequences are listed in the Figure.

In 2020, we further collected fecal sampled of 50 laboratory rabbits in one of the 13 commercial vendors for HEV RNA detection. Six fecal samples were positive for both nested reverse transcription-PCR (RT-nPCR) and Real-Time quantitative PCR (RT-qPCR). Relatively high fecal HEV RNA load was detected ranging from 4.07 × 10^6^ copies/g to 2.45 × 10^7^ copies/g. All samples were successfully sequenced. Phylogenetic analysis of partial ORF2 sequence showed that all sequences belong to HEV-3ra (Figure 1). 

### 2.3. Viral Load in Different Tissues and Urine Samples in Naturally Infected Laboratory Rabbits

**Figure 2:**
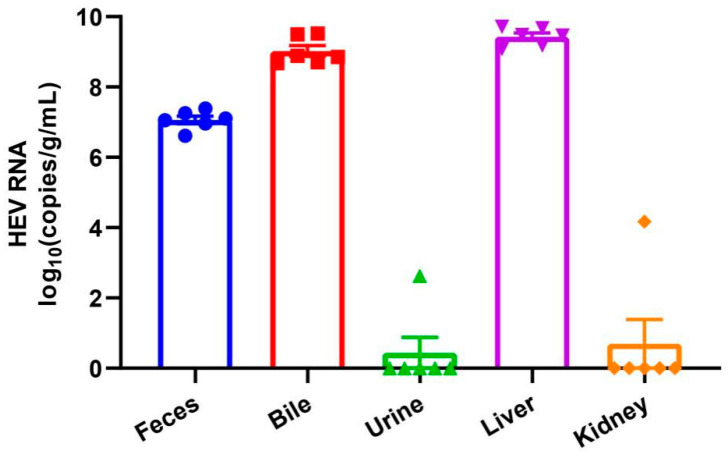
Quantification of HEV RNA in different tissues and samples of laboratory rabbits. The dots indicated the result of individual rabbit.

The six HEV RNA-positive rabbits were euthanized and tissues including liver and kidney, bile and urine samples were collected for HEV RNA quantification. High viral load of HEV RNA was detected in the liver tissues and bile samples (Figure 2). All liver tissues have a viral load exceeding 1 × 10^9^ copies/g. One kidney tissue and one urine sample from different individual rabbit was positive for HEV RNA, with viral load of 1.48 × 10^4^ copies/g and 4.29 × 10^2^ copies/mL, respectively.

### 2.4. Liver Histopathology of the Naturally Infected Laboratory Rabbits

**Figure 3:**
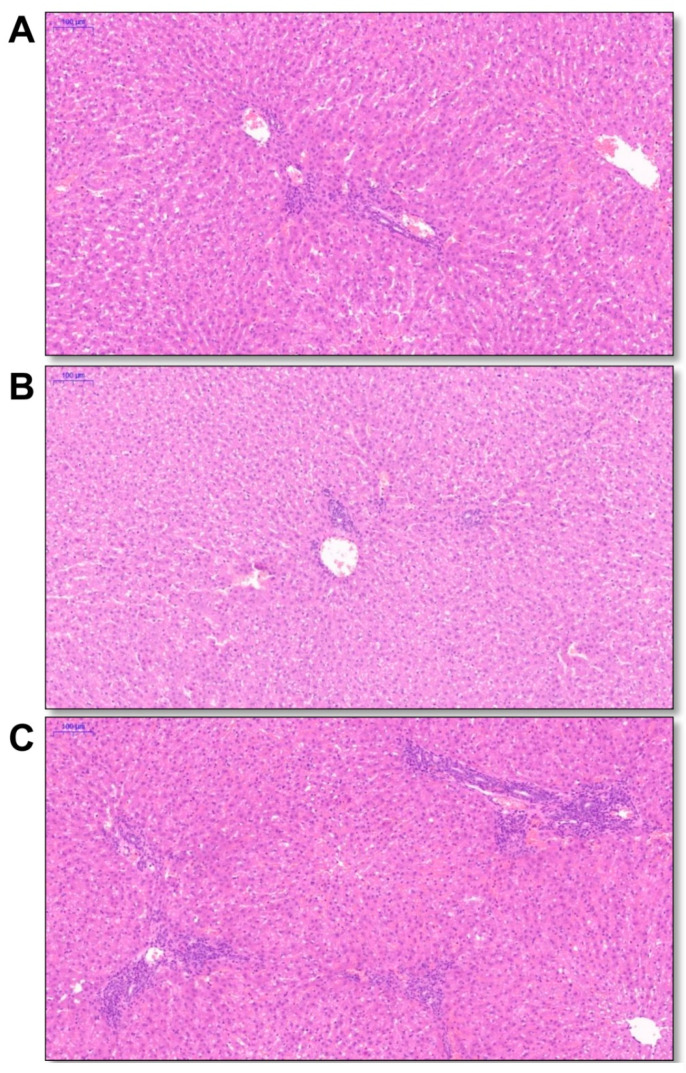
Histopathology of liver tissue in laboratory rabbits. Liver tissues were collected from rabbits No. 35 (**A**), 38 (**B**) and 40 (**C**). Mild-to-moderate inflammatory cell infiltrates were found around the portal area.

To investigate the liver histopathology of the laboratory rabbits with HEV-3ra natural infection, liver tissues were selected from three rabbits (No. 35, 38 and 40) with the highest hepatic viral load for hematoxylin-eosin (HE) staining. Mild-to-moderate infiltration of lymphocytes was found in all liver tissues, indicating inflammation (Figure 3).

## 3. Discussion

In this study, we investigated the prevalence of HEV in laboratory rabbits in a large extent. The result demonstrated that the laboratory rabbits in almost all surveyed vendors (12 out of 13 commercial vendors) experienced a current or past HEV infection event. Six rabbits with current HEV infection were detected in one of the vendors. Sequences obtained from these rabbits all belong to HEV-3ra. The current study highlights the current situation of naturally occurring HEV infection in the laboratory rabbit population. 

In this study, we performed anti-HEV antibodies and HEV Ag (ORF2 proteins) tests by using two different ELISA kits. HEV Ag is a recently discovered novel indicator of HEV infection [25,26] and is now under consideration for further usage in HEV infection diagnosis [27]. The positive result of HEV Ag may indicate active replication of HEV infection. The anti-HEV antibodies we tested are total anti-HEV antibodies. The positive result of ani-HEV antibodies may indicate the rabbit is under recent or past HEV infection. Therefore, the result of the two HEV markers do not necessarily agree. The combination of these two tests we provide a more comprehensive prevalence of HEV in the laboratory rabbit population.

In natural settings, HEV-3ra could infect various breeds of rabbits and the prevalence of anti-HEV and HEV RNA is high [28]. Laboratory rabbits in the USA [23], China [21,22] and Korea [24] have also been reported infected with HEV. The discovery of the present study suggests that HEV is still circulating in the laboratory rabbit population. In 2009, new strains of genotype 3 HEV were identified in rabbits in China [14] and following experiments showed that rabbits may serve as a desirable animal model for human HEV infections study [29], especially in pregnant rabbits, which reproduced the high mortality rate and vertical transmission observed in pregnant women with HEV infection [30]. In addition, further experiments demonstrated that rabbit is also suitable for studying chronic HEV infection [31] and vaccine candidate evaluation [32]. Therefore, a clean background of the laboratory rabbit resource is of great importance for HEV researchers. Rabbits with any positive HEV markers (anti-HEV, HEV-Ag or HEV RNA) may not be qualified for further HEV study [21]. Furthermore, rabbits are widely used in biomedical research; it is unknown that whether laboratory rabbits with positive HEV markers may confound the result of other aspects of biomedical research, such as study of other pathogens or immunology study. 

Recently, several studies reported that HEV-3ra could infect humans and cause chronic infection in immunocompromised patients. The safety of research personnel cannot be ignored. In this study, we detected relatively high level of fecal HEV shedding (10^6^–10^7^ copies/g) of naturally infected laboratory rabbits indicating active replication of HEV in these animals. The result was further confirmed by detecting high viral load of HEV RNA in these rabbits’ bile and liver tissues (approximately 10^9^ copies/g) and inflammation in the liver. Phylogenetic analysis of the six obtained HEV sequences from one of the vendors demonstrated that they all belong to HEV-3ra and clustered closely with the HEV-3ra sequences isolated from rabbits in China. We did not collect serum samples from naturally infected rabbits as previous studies found that fecal virus shedding is longer and more stable in infected rabbits [28]. In a previous study, we found 16 fecal HEV RNA-positive laboratory rabbits and only one of them is viremic [21]. More importantly, HEV is mainly transmitted via fecal-oral route. Therefore, screening HEV RNA in fecal samples can help us better analyzing the risk of potential zoonotic transmission. People are in high risk of HEV exposure when disposing HEV-infected laboratory rabbits. Sufficient and standard precautions should be applied to all research personnel. The transmission of HEV within laboratory rabbit population remains unclear. Since almost all laboratory rabbits were housed individually with separated feeding facility, it was not likely that the infection was acquired via the fecal–oral route. Our previous study demonstrated that vertical transmission existed in experimentally infected pregnant rabbits and HEV RNA could be detected in the offspring (at about 1–2-month-old) [30]. Therefore, the infection in laboratory rabbits may be acquired from their infected mother. Moreover, if the workers at the vendors are infected with HEV, they may transmit the virus to the rabbit during daily animal processing. The exact HEV infection source in the laboratory rabbit population warrants further detection of food source, environmental samples and workers at the vendors. 

In the most recent two decades, HEV4 is the main genotype causing human infection in China [33]. However, several studies reported isolated of HEV3 strains in swine and hepatitis E patients in many provinces in China [34,35,36]. One recent study conducted in Shanghai, reported that 1.2% of the local hepatitis E cases were caused by HEV3 [37]. However, HEV4 is still the dominant strain circulating in swine and human in China. To date, no report found HEV-3ra in humans but the risk exists as HEV-3ra is prevalent in the rabbit population in China.

There are limitations to our study. The sampling size of the current study is still limited and more vendors from other provinces of China should be investigated. Although the rabbits were selected randomly the gender information was not collected. We only detected HEV RNA in one of the vendors and more sequences should be obtained in the future study in different vendors or other provinces in China.

## 4. Materials and Methods

### 4.1. Ethics

The animal experimental protocol was approved by the Committee of Laboratory Animal Welfare and Ethics of the National Institutes for Food and Drug Control [No. 2018(B)006, approved on June 2018] and Peking University Health Science Center (LA2020474, approved on September 2020), China.

### 4.2. Samples

From 2017 to 2019, we performed routine examination of 6-month-old laboratory white rabbits (*Oryctolagus cuniculus*) from 13 commercial vendors in China. Rabbits from each vendor were randomly selected and serum samples were collected from 649 laboratory rabbits during this period. Fifty fecal samples were collected for HEV RNA detection from one of the 13 vendors in 2020. The fecal samples were all diluted with phosphate buffered saline to prepare 10% suspensions and centrifuged at 5000× *g* for 30 min. The supernatants were harvested and immediately stored at −80 °C until use [38]. More detailed information regarding the laboratory rabbits and sampling is shown in Table 1.

Rabbits tested positive for HEV RNA were then purchased for further processing. Liver and kidney tissues were collected immediately after euthanasia. Bile and urine samples were extracted by syringe from the gallbladder and bladder, respectively. All samples were immediately subjected to RNA extraction or stored at −80 °C for later use.

### 4.3. Detection of Anti-HEV Antibodies and HEV Antigen

Total Anti-HEV antibodies and HEV Ag were tested by commercial enzyme-linked immunosorbent assay (ELISA) kits (Wantai, Beijing, China). Signal-to-cutoff (S/CO) values for anti-HEV antibody were calculated and values >1 were considered positive. All experiments were carried out under the manufacturers’ instructions and detailed methods were described in a previous study [39].

### 4.4. Detection of HEV RNA

Approximately 100 mg of the fresh tissue collected was homogenized in 1 mL of TRIzol reagent (Invitrogen, Burlington, ON, Canada) and clarified by centrifugation at 5000× *g* for 15 min at 4 °C and subjected to RNA extraction [33]. RNA was extracted from supernatants, 100 μL of bile or urine using TRIzol reagent. A RT-nPCR with primers targeting partial ORF2 genes of the HEV genome, were used to screen for the presence of HEV RNA [21,38]. HEV-positive samples were commercially sequenced according to the manufacturer’s instructions (Beijing Genomics Institute, Beijing, China) on an automatic DNA sequencer (ABI model 3730 sequencer; Applied Biosystems, Foster City, CA, USA). Standard precautions were taken to avoid PCR contamination as previously described [38], and no false-positive result was observed for negative controls. All sequences were submitted to GenBank with accession numbers MZ032036-MZ032041.

Quantification of HEV RNA was carried out using a commercial One-Step RT-qPCR kit (Promega, Madison, WI, USA) with a previously established in-house method [40]. To quantify the HEV RNA copy number in the sample, serial dilutions of capped HEV RNA (10^9^ to 10^1^ copies) were constructed and used as the standard for quantification of viral genome copy number [31]. The final viral copy number in fecal sample and tissues was adjusted to copies/g, and bile and urine samples to copies/mL.

### 4.5. Phylogenetic Analysis

Nucleotide sequences were assembled and analyzed using MEGA 6.0 [41]. Phylogenetic trees of the six partial HEV-3ra sequences were constructed by the neighbor-joining method and evaluated by the interior branch test method with the aid of the MEGA 6.0 software. One thousand re-samplings of the data were used to calculate the percentage of trees containing each branch. Bootstrap values less than 70% were not shown. All the reference sequences were retrieved from GenBank.

### 4.6. Histopathological Assays

The rabbits’ liver tissues were fixed in 10% neutral buffered formalin and were subsequently embedded in paraffin. Specimens were cut into 4 μm sections. Slides were stained with HE and subjected to histopathological examination [38].

## 5. Conclusions

We demonstrated that past or recent HEV infection existed in laboratory rabbits in Beijing, China, and the results strongly suggested that HEV is widespread in this special animal population. Researchers engage in, not only HEV study, but also other pathogens should be aware of the influence of pre-existing HEV infection on other pathogens infection. Further study should address this issue and the risk of HEV infection in research personnel should be investigated.

## Figures and Tables

**Table 1:** Prevalence of antibodies against hepatitis E virus and antigen in laboratory rabbits, 2017–2019.

Vendor ID	2017			2018			2019		
	No. Animals	Anti-HEV (%)	Ag (%)	No. Animals	Anti-HEV (%)	Ag (%)	No. Animals	Anti-HEV (%)	Ag (%)
BJLRB01	20	10.0	5.0	20	5.0	10.0	20	0	0
BJLRB02	20	0	20.0	20	0	20.0	20	0	0
BJLRB03	20	10.0	25.0	20	0	20.0	20	5.0	0
BJLRB04	20	5.0	20.0	10	0	50.0	0	na	na
BJLRB05	20	5.0	10.0	20	0	5.0	20	5.0	10.0
BJLRB06	20	5.0	0	10	0	0	0	na	na
BJLRB07	20	0 (0/19) ^a^	0	10	10.0	0	10	0 (0/4) ^a^	0
BJLRB08	20	0	0	20	0	5.0	20	0	0
BJLRB09	20	0	5.0	20	0	15.0	20	5.0	0
BJLRB010	20	5.0	5.0	20	0 (0/17) ^a^	10.0	20	0	0
BJLRB011	20	0	20.0	20	10.0 (1/10) ^a^	5.0	20	0	5.0
BJLRB012	19	0	0	10	na	30.0	20	0	0
BJLRB013	20	0	0	0	na	na	0	na	na
Total	259	3.1 (8/258) ^a^	8.5	200	2.3 (4/177) ^a^	13.0	190	1.6 (3/184) ^a^	1.6

^a^ The volume of serum was not sufficient for testing both anti-HEV and Ag and the actual testing number of samples were shown. Ag, antigen; HEV, hepatitis E virus; na, not available.

## Data Availability

The sequences for HEV-3ra have been assigned GenBank accession numbers MZ032036-MZ032041.
